# Estimating the burden of neural tube defects in low– and middle–income countries

**DOI:** 10.7189/jogh.04.010402

**Published:** 2014-06

**Authors:** Annie Lo, Dora Polšek, Simrita Sidhu

**Affiliations:** 1 The University of Edinburgh Medical School, Edinburgh, Scotland, UK; 2 Histology and Embryology Department, School of Medicine, University of Zagreb, Zagreb, Croatia

## Abstract

**Background:**

To provide an estimate for the burden of neural tube defects (NTD) in low– and middle–income countries (LMIC) and explore potential public health policies that may be implemented. Although effective interventions are available to prevent NTD, there is still considerable childhood morbidity and mortality present in LMIC.

**Methods:**

A search of Medline, EMBASE, Global Health Library and PubMed identified 37 relevant studies that provided estimates of the burden of NTD in LMIC. Information on burden of total NTD and specific NTD types was separated according to the denominator into two groups: (i) estimates based on the number of live births only; and (ii) live births, stillbirths and terminations. The data was then extracted and analysed.

**Results:**

The search retrieved NTD burden from 18 countries in 6 WHO regions. The overall burden calculated using the median from studies based on livebirths was 1.67/1000 (IQR = 0.98–3.49) for total NTD burden, 1.13/1000 (IQR = 0.75–1.73) for spina bifida, 0.25/1000 (IQR = 0.08–1.07) for anencephaly and 0.15/1000 (IQR = 0.08–0.23) for encephalocele. Corresponding estimates based on all pregnancies resulting in live births, still births and terminations were 2.55/1000 (IQR = 1.56–3.91) for total NTD burden, 1.04/1000 (IQR = 0.67–2.48) for spina bifida, 1.03/1000 (IQR = 0.67–1.60) for anencephaly and 0.21 (IQR = 0.16–0.28) for encephalocele. This translates into about 190 000neonates who are born each year with NTD in LMIC.

**Conclusion:**

Limited available data on NTD in LMIC indicates the need for additional research that would improve the estimated burden of NTD and recommend suitable aid policies through maternal education on folic acid supplementation or food fortification.

Every year, more than 300 000 children are born with neural tube defects (NTD) [1-6]. NTD are a group of congenital abnormalities that still cause hundreds of thousands of deaths in 0–4 years age group, while similar number of surviving children remain disabled for life [1-6]. One of the Millennium Development Goals initiated by the United Nations was dedicated to reducing global mortality rates of children in this age group. Since 1990, global child mortality has been declining largely due to the focus on communicable diseases, which included the expansion of immunisation programmes, promotion of breast–feeding and increased provision of mosquito bednets in many countries worldwide [2]. This reduction of mortality has led to the neglected causes of child mortality to be exposed, including that of congenital abnormalities [3-6].

NTD are one of the most common presenting birth defects, arising as a result of incomplete closure of the brain or spinal cord in the 3^rd^ and/or 4^th^ week of pregnancy [3]. NTD can be classified as open or closed, depending on whether neural tissues are exposed or covered by skin, respectively. Open NTD are more frequent and include spina bifida, anencephaly and encephalocele. Closed NTD, such as tethered cord syndrome, are less frequent in comparison [4].

The best known risk factor for foetal NTD is maternal folate deficiency, arising from low levels of vitamin B9 (folic acid) [7,8]. Maternal vitamin B12 deficiency has only recently been shown to independently contribute to risk of NTD [9,10]. Additional risk factors for NTD development include a positive family history, smoking and indoor air pollution from coal and biomass heating used predominantly in developing countries [11-15]. Moreover, NTD are related to maternal socio–economic status, education, area of residence, and maternal nutrient deficiency or obesity [16,17].

NTD can be identified through simple prenatal testing using ultrasound imaging or maternal serum alpha–fetoprotein (MSAFP) level screening [18]. Abnormal elevation of MSAFP is a relatively specific and sensitive test for detection of NTD [18]. The abnormal presence of acetylcholinesterases (AChE) in amniotic fluid determined through amniocentesis can also be used for screening of NTD. However, a higher–than–normal test result is often not diagnostic and further evaluation should always be undertaken [19,20].

Since the discovery of folic acid as an effective intervention for prevention of neural tube defects [21], many countries have recommended folic acid intake before conception and during pregnancy. However, the dramatic 20% decrease in NTD birth burden after mandatory folic acid fortification (FAF) of enriched products in the US in 1998 showed that there may be more practical ways to administer this intervention [22]. Since this example, many countries such as Chile, Saudi Arabia and South Africa have implemented similar measures to staple food [23-25]. Despite folic acid being a well–known, cost–effective intervention, many developing countries continue to have either ineffective or no policy to increase maternal uptake of folic acid to prevent NTD.

The aims and objectives of this systematic review were:

1. To provide an estimate of NTD burden in LMIC by systematically reviewing literature available in public domain;

2. To examine and discuss the significance of these findings and consider clinical and cost–effective interventions and health policies with regards to NTD.

## METHODS

A systematic literature review was conducted to search for published literature regarding population–based NTD burden estimates in LMIC, through the use of electronic databases: Medline, Embase, Global Health Library and PubMed. Potential further data were searched for on Google Scholar and by crosschecking reference lists from review articles. The search used Medical Subject Headings (MeSH) and key words for the burden of NTD in LMIC, as outlined by the World Bank. Limits of “human” and “2000–current” were used to obtain the most up to date NTD burden information. The last searches of the four databases were conducted on 6 February 2013. Search terms for Medline are shown in **Table 1** and were modified for other databases as required.

**Table 1:** Search terms for Medline

1.	exp Developing Countries/
2.	Developing countr*.tw
3.	(developing adj3 countr*).tw
4.	africa/ or africa, northern/ or algeria/ or egypt/ or libya/ or morocco/ or tunisia/ or “africa south of the sahara”/ or africa, central/ or cameroon/ or central african republic/ or chad/ or congo/ or “democratic republic of the congo”/ or equatorial guinea/ or gabon/ or africa, eastern/ or burundi/ or djibouti/ or eritrea/ or ethiopia/ or kenya/ or rwanda/ or somalia/ or sudan/ or tanzania/ or uganda/ or africa, southern/ or angola/ or botswana/ or lesotho/ or malawi/ or mozambique/ or namibia/ or south africa/ or swaziland/ or zambia/ or zimbabwe/ or benin/ or burkinafaso/ or cape verde/ or cote d'ivoire/ or gambia/ or ghana/ or guinea/ or guinea–bissau/ or liberia/ or mali/ or mauritania/ or niger/ or nigeria/ or senegal/ or sierra leone/ or togo/ or americas/ or caribbean region/ or west indies/ or “antigua and barbuda”/ or bahamas/ or barbados/ or cuba/ or dominica/ or dominican republic/ or grenada/ or guadeloupe/ or haiti/ or jamaica/ or martinique/ or netherlandsantilles/ or puertorico/ or “saint kitts and nevis”/ or saint lucia/ or “saint vincent and the grenadines”/ or “trinidad and tobago”/ or central america/ or belize/ or costa rica/ or el salvador/ or guatemala/ or honduras/ or nicaragua/ or panama/ or panama canal zone/ or latinamerica/ or mexico/ or south america/ or argentina/ or bolivia/ or brazil/ or chile/ or colombia/ or ecuador/ or frenchguiana/ or guyana/ or paraguay/ or peru/ or suriname/ or uruguay/ or venezuela/ or kazakhstan/ or kyrgyzstan/ or tajikistan/ or turkmenistan/ or uzbekistan/ or borneo/ or brunei/ or cambodia/ or east timor/ or indonesia/ or laos/ or malaysia/ or mekong valley/ or myanmar/ or philippines/ or thailand/ or vietnam/ or bangladesh/ or bhutan/ or india/ or sikkim/ or middle east/ or afghanistan/ or iran/ or iraq/ or israel/ or jordan/ or lebanon/ or saudiarabia/ or syria/ or turkey/ or united arab emirates/ or yemen/ or nepal/ or pakistan/ or srilanka/ or far east/ or china/ or tibet/ or “democratic people's republic of korea”/ or mongolia/ or taiwan/ or albania/ or lithuania/ or bosnia–herzegovina/ or bulgaria/ or “macedonia (republic)”/ or moldova/ or montenegro/ or romania/ or russia/ or bashkiria/ or dagestan/ or moscow/ or siberia/ or serbia/ or ukraine/ or armenia/ or atlantic islands/ or azores/ azerbaijan/ or “georgia (republic)”/ or comoros/ or madagascar/ or mauritius/ or seychelles/ or vanuatu/ or micronesia/ or palau/ or expsamoa/ or americansamoa/ or “independent state of samoa”/ or tonga/
5.	exp neural tube defects/ or anencephaly/ or arnold–chiari malformation/ or encephalocele/ or meningocele/ or meningomyelocele/ or “pentalogy of cantrell”/ or exp spinal dysraphism/ or nervous system malformation
6.	“neural tube defect”.tw
7.	“neural tube defects”.tw
8.	NTD.tw
9.	exp Prevalence/
10.	prevalen*.tw
11.	1 or 2 or 3 or 4
12.	5 or 6 or 7 or 8
13.	9 or 10
14.	11 and 12 and 13
15.	Limit 14 to (humans and yr = ”2000 –Current”)

### Study selection

The inclusion criteria for relevant papers included population or hospital based studies conducted in LMIC, which were geographically defined taking into account both the World Health Organization's and the World Bank's classification and treating any discrepancies in an inclusive, rather than exclusive way. The studies needed to have clearly expressed NTD burden showing a denominator, published between 2000 and 2013. The searches were limited to the period after the year 2000 in order to generate an estimate that is reflective of reasonably recent NTD trends. No limit on language and publication type was set. Keeping in mind that many babies with NTD are stillborn or terminated through miscarriages and abortions, we decided to include studies with live births, stillbirths and terminations as a separate body of evidence, in addition to studies that used live births–based denominators to report the burden of NTD.

Studies conducted solely in specialist hospital units were excluded, as they are likely to report a burden enriched for severe cases that would not be representative of the general population. Studies with incomplete data or where NTD burden could not be calculated were also excluded.

### Data extraction

For the 37 retained studies, relevant data were extracted and compiled into Microsoft Excel spreadsheets. Data including authors, country, study size and diagnostic criteria for specific NTD type and total NTD cases were extracted. Types of NTD included spina bifida, myelomeningocele, meningocele, anencephaly, encephalocele and “other NTD types”. Burden was expressed using the number of cases observed and a total sample of live births (or, alternatively, a total sample of live births, stillbirths and terminations).

### Data analysis

When the number of affected children was not specifically provided in the study, it was calculated with the sample population using the following equation:

Estimated NTD burden = Number of observed NTD cases/Sample size (eg, number of live births) × 1000

The estimates of the burden provided in the retained studies were separated into two categories: those in which the denominator was based on the number of live births, and the other group in which live births, stillbirths and terminations were all included. Wherever this information was available for both categories, figures were separately added to the respective groups. The median estimate of the NTD burden and the inter–quartile range (IQR) for all LMIC regions was then determined, based on the retained 37 studies. Eventually, the median was multiplied by the number of livebirths in LMIC in the year 2010, according to UN Population Division's estimates (www.un.org/esa/population/‎), to determine the absolute number of NTD cases that has been introduced to the LMIC in 2010.

## RESULTS

**Figure 1:**
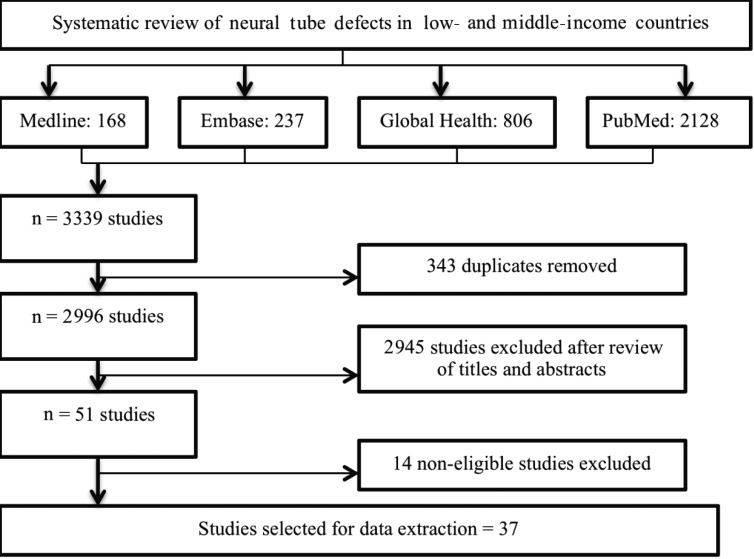
A summary of the process of literature search.

A review of relevant databases performed independently by two researchers (AL and SS) identified a total of 3339 studies, but only 37 satisfied all criteria for inclusion (as shown in **Figure 1**). Of the retained studies, 20 reported NTD rates in live births only, 14 reported rates in live births, stillbirths and terminations combined, and 3 studies reported both.

The median sample size from all reviewed papers was 36 331, which corresponded well to a typical study size. The median sample population in studies based on live births was 35 974, compared to 49 534 in studies based on live births, stillbirths and terminations.

The search retrieved NTD burden from 18 countries in 6 WHO regions (**Table 2**). The overall burden calculated using the median from studies based on live births was 1.67/1000 (IQR = 0.98–3.49) for total NTD burden (**Table 3**** and ****4**), 1.13/1000 (IQR = 0.75–1.73) for spina bifida (**Table 3**** and ****5**), 0.25/1000 (IQR = 0.08–1.07) for anencephaly (**Table 3**** and ****6**) and 0.15/1000 (IQR = 0.08–0.23) for encephalocele (**Table 3**** and ****7**).Corresponding estimates based on all pregnancies resulting in live births, still births and terminations were 2.55/1000 (IQR = 1.56–3.91) for total NTD burden (**Table 3**** and ****8**), 1.04/1000 (IQR = 0.67–2.48) for spina bifida (**Table 3**** and ****9**), 1.03/1000 (IQR = 0.67–1.60) for anencephaly (**Table 3**** and ****10**) and 0.21 (IQR = 0.16–0.28) for encephalocele (**Table 3**** and ****11**). This translates into about 190 000 neonates who are born each year with NTD in LMICs.

**Table 2:** Distribution of retained studies by WHO regions

		Number of studies		
**WHO region**	**Country**	**Total studies**	**Live births only**	**Live births, stillbirths & terminations**
Western Pacific	China	6	1	5
	Malaysia	1	0	1
South East Asia	India	3	2	1
	Pakistan	1	1	0
	Thailand	1	1	0
Eastern Mediterranean	Jordan	2	2	0
	Saudi Arabia	2	2	0
	Iran	4	1	3
Europe	Azerbaijan	1	0	1
	Russia	1	1	0
	Ukraine	1	0	1
	Turkey	3	2	1
Africa	Cameroon	1	1	0
	South Africa	1	1	0
Americas	Brazil	3	2	1
	Colombia	1	1	0
	Peru	1	1	0
	Chile	4	1	3

**Table 3:** A summary of estimates of the burden of neural tube defects and its sub–types from 37 retained studies from low and middle–income countries

Studied outcome	Denominator	Number of Studies	Median (per 1000)	Inter–quartile range (per 1000)	Minimum(per 1000)	Maximum (per 1000)
All neural tube defects	LB	23	1.67	0.98–3.49	0.50	12.41
	LB+SB+TP	17	2.55	1.56–3.91	0.86	19.94
Spina bifida	LB	14	1.13	0.75–1.73	0.38	5.90
	LB+SB+TP	15	1.04	0.67–2.48	0.35	5.81
Anencephaly	LB	13	0.25	0.08–1.07	0.01	11.33
	LB+SB+TP	16	1.03	0.67–1.60	0.30	8.26
Encephalocele	LB	9	0.15	0.08–0.23	0.03	0.39
	LB+SB+TP	13	0.21	0.16–0.28	0.07	2.65

LB – live births; LB+SB+TP – live births, stillbirths and terminated pregnancies

**Table 4:** Studies that reported rates for total NTD burden based on live births only

Author and reference	Sample size	Cases	Rate (per 1000 live births)
Amarin et al. [26]	61 447	16	0.95
Aqrabawi [11]	5088	33	6.50
Asindi et al. [27]	82 176	64	0.78
Bademci et al. [28]	5499	17	3.09
Behrooz et al. [29]	13 262	56	4.22
Chen et al. [30]	26 599	48	1.80
Cherian et al. [31]	1218	10	8.21
Cortes et al. [32]	59 627	67	1.12
Costa et al. [33]	9386	11	1.17
Gu et al. [13]	6420	25	3.89
Hertrampf et al. [34]	117 740	114	0.97
Kaur et al. [35]	7400	5	0.68
Khattak et al. [36]	5560	69	12.41
Mandiracioglu et al. [37]	36 331	56	1.54
Njamnshi et al. [38]	52 710	98	1.86
Pachajoa et al. [39]	32 995	55	1.67
Petrova et al. [40]	141 159	298	2.11
Pacheco et al. [41]	24 964	124	4.97
Ricks et al. [42]	35 974	72	2.00
Safdar et al. [24]	33 489	42	1.25
Sayed et al. [25]	46 021	45	0.98
Wasant et al. [43]	180 000	114	0.63
Yuskiv et al. [33]	75 609	38	0.50

**Table5:** Studies that reported rates for the burden of spina bifida based on live births only

Author and reference	Sample size	Cases	Rate(per 1000 live births)
Aqrabawi[11]	5088	30	5.90
Asindi et al. [27]	82 176	46	0.56
Bademci et al. [28]	5499	11	2.00
Behrooz et al. [29]	13 262	23	1.73
Cherian et al. [31]	1218	6	4.93
Costa et al. [44]	9386	7	0.75
Khattak et al. [36]	5560	5	0.90
Mandiracioglu et al. [37]	36 331	43	1.18
Njamnshi et al. [38]	52 710	65	1.23
Petrova et al. [40]	141 159	147	1.04
Ricks et al. [42]	35 974	62	1.72
Safdar et al. [24]	33 489	36	1.07
Sayed et al. [25]	46 021	25	0.54
Yuskiv et al. [33]	75 609	29	0.38

**Table 6:** Studies that reported rates for the burden of anencephaly based on live births only

Author and reference	Sample size	Cases	Rate (per 1000 live births)
Asindi et al. [27]	82 176	3	0.04
Behrooz et al. [29]	13 262	30	2.26
Cherian et al. [31]	1218	3	2.46
Costa et al. [44]	9386	1	0.11
Khattak et al. [36]	5560	63	11.33
Mandiracioglu et al. [45]	36 331	4	0.11
Njamnshi et al. [38]	52 710	4	0.08
Petrova et al. [40]	141 159	151	1.07
Ricks et al. [42]	35 974	10	0.28
Safdar et al. [24]	33 489	1	0.03
Sayed et al. [25]	46 021	17	0.37
Wasant et al. [43]	180 000	45	0.25
Yuskiv et al. [33]	75 609	1	0.01

**Table 7:** Studies that reported rates for the burden of encepalocele based on live births only

Author and reference	Sample size	Cases	Rate (per 1000 live births)
Aqrabawi [11]	5088	2	0.39
Asindi et al. [27]	82 176	15	0.18
Behrooz et al. [29]	13 262	3	0.23
Costa et al. [44]	9386	3	0.32
Mandiracioglu et al. [45]	36 331	1	0.03
Njamnshi et al. [38]	52 710	5	0.09
Safdar et al. [24]	33 489	5	0.15
Wasant et al. [43]	180 000	14	0.08
Yuskiv et al. [33]	75 609	3	0.03

**Table 8:** Studies that reported rates for total NTD burden based on live births, stillbirths and terminations of pregnancy

Author and reference	Sample size	Cases	Rate (per 1000 live births stillbirths and terminations)
Aguiar et al. [46]	18 807	89	4.73
Cortes et al. [32]	60 072	94	1.56
Cortes et al. [12]	486 779	419	0.86
Dai et al. [46]	2 281 616	2873	1.30
Golalipour et al. [47]	37 951	109	2.87
Golalipour et al. [12]	30 639	78	2.55
Golalipour et al. [48]	49 534	194	3.91
Gu et al. [13]	6420	128	19.94
Li et al. [10]	11 534	159	13.79
Liu et al. [49]	99 888	122	1.22
Mahadevan et al. [45]	54 738	310	5.66
Nazer et al. [50]	434 624	740	1.70
Noraihan et al. [51]	34 109	37	1.08
Onrat et al. [52]	8631	31	3.59
Rad et al. [53]	14 121	117	2.57
Yuskiv et al. [33]	75 928	159	2.09
Zhang et al. [54]	62 373	126	2.02

**Table 9:** Studies that reported rates for the burden of spina bifida based on live births, stillbirths and terminations of pregnancy

Author and reference	Sample size	Cases	Rate (per 1000 live births, stillbirths and terminations)
Cortes et al. [32]	60 072	46	0.77
Cortes et al. [12]	486 779	204	0.42
Golalipour et al. [47]	37 951	62	1.63
Golalipour et al. [12]	30 639	39	1.27
Gu et al. [13]	6420	25	3.89
Li et al. [10]	11 534	67	5.81
Liu et al. [49]	99 888	59	0.59
Mahadevan et al. [45]	54 738	170	3.11
Noraihan et al. [51]	34 109	12	0.35
Onrat et al. [52]	8631	9	1.04
Yuskiv et al. [33]	75 928	64	0.84
Aguiar et al. [55]	18 807	47	2.49
Dai et al. [46]	2 281 616	1369	0.60
Nazer et al. [50]	434 624	374	0.86
Rad et al. [53]	14 121	35	2.48

**Table 10:** Studies that reported rates for the burden of anencephaly based on live births, stillbirths and terminations of pregnancy

Author and reference	Sample size	Cases	Rate (per 1000 live births, stillbirths and terminations)
Aguiar et al. [55]	18 807	24	1.28
Cortes et al. [32]	60 072	37	0.62
Cortes et al. [12]	486 779	147	0.30
Dai et al. [46]	2 281 616	1140	0.50
Golalipour et al. [47]	37 951	43	1.40
Golalipour et al. [12]	30 639	35	0.92
Golalipour et al. [48]	49 534	56	1.13
Gu et al. [13]	6420	53	8.26
Li et al. [10]	11 534	75	6.50
Liu et al. [49]	99 888	42	0.42
Mahadevan et al. [45]	54 738	98	1.80
Nazer et al. [50]	434 624	311	0.72
Noraihan et al. [51]	34 109	25	0.73
Onrat et al. [52]	8631	12	1.39
Rad et al. [53]	14 121	78	5.52
Yuskiv et al. [33]	75 928	62	0.82

**Table 11:** Studies that reported rates for the burden of encephalocele based on live births, stillbirths and terminations of pregnancy

Author and reference	Sample size	Cases	Rate (per 1000 live births stillbirths and terminations)
Aguiar et al. [55]	18 807	5	0.27
Cortes et al. [32]	60 072	11	0.18
Dai et al. [46]	2 281 616	365	0.16
Golalipour et al. [47]	37 951	4	0.11
Golalipour et al. [12]	30 639	4	0.13
Gu et al. [13]	6420	17	2.65
Li et al. [10]	11 534	17	1.47
Liu et al. [49]	99 888	7	0.07
Mahadevan et al. [45]	54 738	36	0.66
Nazer et al. [50]	434 624	91	0.21
Onrat et al. [52]	8631	2	0.23
Rad et al. [53]	14 121	4	0.28
Yuskiv et al. [33]	75 928	12	0.16

As expected, when comparing IQRs as the robust predictions, overall NTD burden estimates were found to be higher in the live births, stillbirths and terminations group in comparison to studies that included only live births, while spina bifida was the most commonly reported NTD type. Moreover, there is internal consistency in the presented estimates, because the sum of the specific NTD types always fits into the “envelope” of all NTD.

## DISCUSSION

This systematic literature review aimed to examine the burden of NTD in LMIC. It is, to our knowledge, the first study to quantitatively estimate the total NTD burden in LMIC. As such, the burden estimates can be successfully used in the much–needed preventive policy development in LMIC with high risk of NTD.

The results from the 37 selected studies [10-13,23-55] suggest that NTD burden is approximately twice as high, if not higher, in LMIC than in high–income countries [56-58]. The findings from live birth–only studies showed that the median total NTD burden is 1.67 per 1000 live births, although there were reports of significantly higher values, with a maximum burden as high as 12.41/1000. The overall median is greater in studies where live births, stillbirths and terminations were taken into account, where the burden is 2.55 per 1000 and maximum reported burden of 19.94/1000. This is expected, as a considerable proportion of NTD result in stillbirths and terminations [59,60].

Significant discrepancies between reported burdens from the same country were sometimes observed. These differences were attributed to different study settings, for example in rural and urban India [31,35,45], or different time periods as seen in two studies from Jordan [11,26]. Extremely high burden of NTD of 13.79 and 19.94 was observed in two studies from China, although the samples were rather small, indicating a possible selection bias [10,13].

Regardless of the progress in control of NTDs observed in high–income countries, NTD continue to be a problem of significant public health impact in LMIC. NTD have detrimental physical and emotional effects on the affected children and their caregivers, and may present a life–long important and often insurmountable economic problem, especially to poor families [52]. The cost of raising a child with spina bifida from birth to 18 years of age in Chile was estimated to be around US$ 120 000 [34]. These expenses, apart from causing individual deprivation, are a significant economic burden on the level of the whole society, causing a vicious circle of poverty in the LMIC.

Hundreds of thousands of live born babies are affected by NTD in LMIC, which remain an important and preventable cause of morbidity and mortality. Thus, effective policies for prevention are vital to reduce the burden of NTD on individuals and on society. Up to now, more than 59 countries have committed to mandatory fortification programmes [59,61,62]. However, many LMIC still have ineffective recommendations and policies towards folic acid uptake. Some countries have recommended the improvement of daily diet and folic acid supplement use, but do not have a mandatory policy [59,61,62]. Recommendation provides a good starting point for reducing NTD burden in LMIC. However, many households in LMIC may not be able to afford folic acid supplementation throughout pregnancy [63]. As shown by the example from the US where NTD burden had fallen by 20% after mandatory fortification, recommendation alone, even without the economic constraint, is not likely to provide a feasible and effective solution [22].Interestingly, survey conducted in the UK found that there was only a marginal increase in folic acid intake in women who were planning pregnancy [64]. Additionally, around half of all pregnancies in the US are unexpected [58,62], and this figure may be even higher in LMIC where there may be limited availability of contraception.

Despite obvious benefits, before promoting folic acid fortification, many factors must be considered. Currently no country in the European Union has compulsory fortification schemes due to risk consideration and campaigns against ‘mass medication’ [65,66]. Safety, ethics and economic feasibility of a FAF programme must be taken into account before implementing such a policy, especially on a whole–country level. Nevertheless, current high burden of NTD in LMIC stresses the need for a comprehensive prevention program.

For consistent and reliable estimates on burden of NTD, it is important to set up vital and birth registration documentation programs in countries that lack coherent information on NTD burden. Not only will this aid in the prevention and treatment of NTD, but it will also enable policy makers to monitor the benefits of implemented prevention programs. This may be particularly important for countries in the African WHO region, where a high NTD burden is expected, but from which only a few studies have been published [67,68].

The reported NTD burden was estimated based on a limited number of available studies, some with very variable sample sizes that differed in inclusion of stillbirths and terminated births in the study design. We could not use meta–analysis, because studies came from such heterogeneous contexts that we didn't feel it was justified to present anything beyond simple median and IQR in this initial estimate. This is partly because not all studies adhered to ICD–10 classification of NTD and were not uniformly conducted regarding method of diagnosis and reporting of NTD type, enabling potential over– or under–estimation of NTD burden through misdiagnosis. Also, the technical restrictions of accounting for all stillbirths and terminations in the examined studies limited the precision of our estimated burden in that population [68].

Finally, the data was available from studies conducted in only 18 countries, implying that the studied sample is unlikely to be representative of all the LMIC globally. Regardless of these significant limitations, it is our opinion that the estimated burdens reported in the results provide useful data for initial assessment of NTD burden in LMIC. An increase in high quality research on NTD, especially with regards to gender and geographical regions, should be prioritised to allow more accurate NTD estimates. This would make the burden of the problem easier to estimate in a more credible way, and allow effective planning of prevention and intervention to minimise the risks for NTD.
